# Diethyl 2,3-dihydro­thieno[3,4-*b*]-1,4-dioxine-5,7-dicarboxyl­ate

**DOI:** 10.1107/S1600536808000937

**Published:** 2008-01-18

**Authors:** Katsuhiko Ono, Masaaki Tomura, Katsuhiro Saito

**Affiliations:** a Department of Materials Science and Engineering, Nagoya Institute of Technology, Gokiso, Showa-ku, Nagoya 466-8555, Japan; b Institute for Molecular Science, Myodaiji, Okazaki 444-8585, Japan

## Abstract

The title compound, C_12_H_14_O_6_S, is a dicarboxylic acid diethyl ester of 3,4-ethyl­enedioxy­thio­phene, which is a component of electrically conductive poly(3,4-ethyl­enedioxy­thio­phene) (PEDOT). The ethyl­ene group is disordered over two sites with occupancy factors 0.64 and 0.36. Both the carbonyl groups are coplanar with the thio­phene ring. The mol­ecules form centrosymmetric dimers with an *R*_2_^2^(12) coupling by inter­molecular C—H⋯O hydrogen bonds [3.333 (5) Å] at the ethoxy­carbonyl groups. The dimer units are arranged to form a ribbon-like mol­ecular sheet.

## Related literature

The title compound was synthesized as a precursor of 3,4-ethyl­enedioxy­thio­phene, which is polymerized to afford PEDOT (Groenendaal *et al.*, 2000 ▶; Pei *et al.*, 1994 ▶). Synthetic methods for the title compound have been reported by: Coffey *et al.* (1996 ▶); Kumar *et al.* (1998 ▶); Zong *et al.* (2002 ▶); Caras-Quintero & Bäuerle (2002 ▶). For literature on related mol­ecular structures, including a 3,4-ethyl­enedioxy­thio­phene ring system, see: Sotzing *et al.* (1996 ▶); Abboud *et al.* (1998 ▶); Kumar *et al.* (1998 ▶). For related literature, see: Bernstein *et al.* (1995 ▶); Allen *et al.* (1987 ▶).
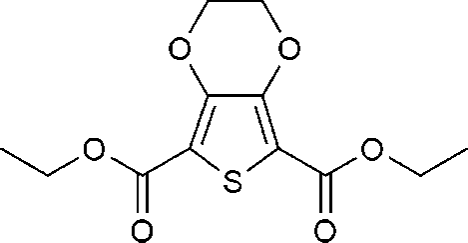


## Experimental

### 

#### Crystal data

C_12_H_14_O_6_S
*M* *_r_* = 286.30
Triclinic, 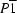

*a* = 4.6805 (8) Å
*b* = 8.3673 (17) Å
*c* = 17.351 (3) Å
α = 94.294 (7)°
β = 92.024 (9)°
γ = 105.641 (9)°
*V* = 651.4 (2) Å^3^
*Z* = 2
Mo *K*α radiation
μ = 0.27 mm^−1^
*T* = 295 (1) K
0.60 × 0.10 × 0.08 mm

#### Data collection

Rigaku/MSC Mercury CCD diffractometer
Absorption correction: none
5181 measured reflections
2899 independent reflections
2300 reflections with *I* > 2σ(*I*)
*R*_int_ = 0.036

#### Refinement

*R*[*F* ^2^ > 2σ(*F* ^2^)] = 0.069
*wR*(*F* ^2^) = 0.176
*S* = 1.11
2899 reflections
193 parameters
H-atom parameters constrained
Δρ_max_ = 0.55 e Å^−3^
Δρ_min_ = −0.26 e Å^−3^

### 

Data collection: *CrystalClear* (Rigaku/MSC, 2001 ▶); cell refinement: *CrystalClear*; data reduction: *CrystalClear*; program(s) used to solve structure: *SHELXS97* (Sheldrick, 2008 ▶); program(s) used to refine structure: *SHELXL97* (Sheldrick, 2008 ▶); molecular graphics: *PLATON* (Spek, 2003 ▶) and *Mercury* (Macrae *et al.*, 2006 ▶); software used to prepare material for publication: *SHELXL97*.

## Supplementary Material

Crystal structure: contains datablocks global, I. DOI: 10.1107/S1600536808000937/hg2368sup1.cif

Structure factors: contains datablocks I. DOI: 10.1107/S1600536808000937/hg2368Isup2.hkl

Additional supplementary materials:  crystallographic information; 3D view; checkCIF report

## Figures and Tables

**Table 1:** Hydrogen-bond geometry (Å, °)

*D*—H⋯*A*	*D*—H	H⋯*A*	*D*⋯*A*	*D*—H⋯*A*
C9—H9*B*⋯O3^i^	0.96	2.66	3.333 (5)	127
C9—H9*A*⋯O3^ii^	0.96	2.62	3.523 (7)	157
C6*B*—H6*B*1⋯O5^iii^	0.97	2.68	3.233 (12)	117

Symmetry codes: (i) 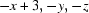; (ii) 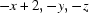; (iii) 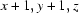.

**Fig. 1.**
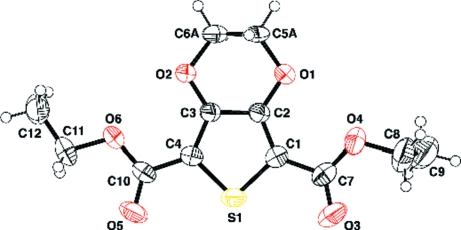
The molecular structure of (I), with atom labels and 50% probability displacement ellipsoids for non-H atoms and H atoms are shown as small spheres of arbitrary radii. The disordered atoms (C5B and C6B) are omitted for clarity.

**Fig. 2.**
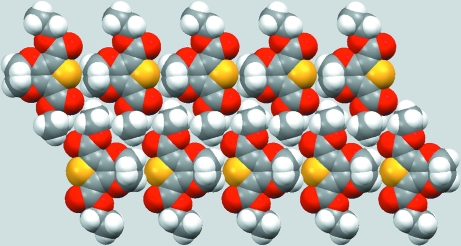
The packing diagram of (I), ribbon-like molecular sheet.

**Fig. 3.**
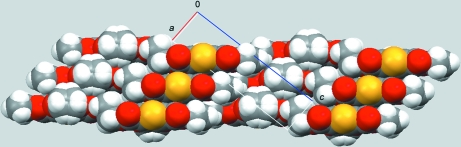
The packing diagram of (I), packing mode of molecular sheets.

## supplementary crystallographic information

### Comment

The title compound (I) has been prepared as a precursor of 3,4-ethylenedioxythiophene (Coffey *et al., *1996; Kumar *et al., *1998; Zong *et al., *2002; Caras-Quintero & Bäuerle, 2002), which is polymerized by oxidizing agents to afford poly(3,4-ethylenedioxythiophene) (PEDOT). PEDOT shows high electrical conductivities and high stabilities in the oxidized states. Furthermore, the thin films of oxidized PEDOT are almost transparent. Therefore, these are used for organic electrodes in the study of electronic devices (Groenendaal *et al., *2000; Pei *et al., *1994). With regard to the hole-transporting abilities, the arrangement of 3,4-ethylenedioxythiophene units in film has attracted considerable attention. A few crystal structures including a 3,4-ethylenedioxythiophene ring system were reported (Sotzing *et al., *1996; Abboud *et al., *1998; Kumar *et al., *1998). In this paper, we report the crystal structure of compound (I) that is a dicarboxylic acid diethyl ester of 3,4-ethylenedioxythiophene.

The compound (I) crystallizes in the *P*1 space group. The molecular structure is shown in Fig. 1. The ethylene moiety is disordered over two sites (O1—C5A—C6A—O2 and O1—C5B—C6B—O2) with occupancies of 0.36:0.64. The bond lengths and angles are all within expected ranges (Allen *et al.*, 1987). Both the carbonyl moieties are planar to the thiophene ring. The molecules form a centrosymmetric dimer with a graph-set motif (Bernstein *et al.*, 1995) of *R*_2_^2^(12) by intermolecular C–H···O hydrogen bonds at the ethoxycarbonyl groups [C9–H9B···O3(–*x* + 3, –*y*, –*z*): 3.333 (5) Å]. The dimer units are arranged to form a ribbon-like molecular sheet along the *b* axis, as shown in Fig. 2. The ribbon-like molecular sheets stack to form a layer structure (Fig. 3).

### Experimental

The title compound (I) was prepared as follows: A solution of diethyl 3,4-dihydroxythiophene-2,5-dicarboxylate (3.12 g, 12 mmol) and caesium fluoride (7.26 g, 48 mmol) in dry acetonitrile (200 ml) was stirred for 1 h under nitrogen. After addition of a solution of ethylene di(*p*-toluenesulfonate) (5.55 g, 15 mmol) in dry acetonitrile (100 ml), the reaction mixture was refluxed for 48 h. The reaction mixture was filtered and the precipitate was washed with acetonitrile. The filtrate was concentrated and the residue was chromatographed on alumina gel (CH_2_Cl_2_) and silica gel (CH_2_Cl_2_) to afford the compound of (I) (2.38 g, 69%) as colorless needles. Physical data for (I): m.p. 424–425 K; IR (KBr, cm^-1^) 2998, 1698, 1454, 1377, 1302, 1098; ^1^H NMR (CDCl_3_, δ p.p.m): 1.37 (t, J = 7.1 Hz, 6H), 4.35 (q, J = 7.1 Hz, 4H), 4.40 (s, 4H); ^13^C NMR (CDCl_3_, δ p.p.m): 14.2, 61.3, 64.7, 111.8, 144.9, 160.7; MS (EI): m/*z* 286 (*M*^+^), 241, 213, 169. Anal. Calcd for C_12_H_14_O_6_S: C, 50.34; H, 4.93. Found: C, 50.50; H, 4.96. Colorless crystals of (I) suitable for X-ray analysis were obtained from a methanol solution.

### Refinement

All the H atoms were placed in geometrically calculated positions, with C—H = 0.97 (methylene) and 0.96 (methyl) Å and *U*_iso_(H) = 1.2*U*_eq_(C) (methylene) and 1.5*U*_eq_(C) (methyl), and refined using a riding model.

### Crystal data

**Table:**

C_12_H_14_O_6_S	*Z* = 2
*M**_r_* = 286.30	*F*_000_ = 300
Triclinic, *P*1	*D*_x_ = 1.460 Mg m^−^^3^
Hall symbol: -P 1	Mo *K*α radiation λ = 0.71070 Å
*a* = 4.6805 (8) Å	Cell parameters from 1654 reflections
*b* = 8.3673 (17) Å	θ = 3.3–27.5º
*c* = 17.351 (3) Å	µ = 0.27 mm^−^^1^
α = 94.294 (7)º	*T* = 295 (1) K
β = 92.024 (9)º	Plate, colorless
γ = 105.641 (9)º	0.60 × 0.10 × 0.08 mm
*V* = 651.4 (2) Å^3^	

### Data collection

**Table:**

Rigaku/MSC Mercury CCD diffractometer	2300 reflections with *I* > 2σ(*I*)
Monochromator: Graphite Monochromator	*R*_int_ = 0.036
Detector resolution: 14.6199 pixels mm^-1^	θ_max_ = 27.5º
*T* = 295(1) K	θ_min_ = 3.3º
φ and ω scans	*h* = −4→6
Absorption correction: none	*k* = −10→10
5181 measured reflections	*l* = −22→19
2899 independent reflections	

### Refinement

**Table:**

Refinement on *F*^2^	Secondary atom site location: difference Fourier map
Least-squares matrix: full	Hydrogen site location: inferred from neighbouring sites
*R*[*F*^2^ > 2σ(*F*^2^)] = 0.069	H-atom parameters constrained
*wR*(*F*^2^) = 0.176	*w* = 1/[σ^2^(*F*_o_^2^) + (0.0793*P*)^2^ + 0.337*P*] where *P* = (*F*_o_^2^ + 2*F*_c_^2^)/3
*S* = 1.11	(Δ/σ)_max_ < 0.001
2899 reflections	Δρ_max_ = 0.55 e Å^−^^3^
193 parameters	Δρ_min_ = −0.26 e Å^−^^3^
Primary atom site location: structure-invariant direct methods	Extinction correction: none

### Special details

**Table:**

Geometry. All e.s.d.'s (except the e.s.d. in the dihedral angle between two l.s. planes) are estimated using the full covariance matrix. The cell e.s.d.'s are taken into account individually in the estimation of e.s.d.'s in distances, angles and torsion angles; correlations between e.s.d.'s in cell parameters are only used when they are defined by crystal symmetry. An approximate (isotropic) treatment of cell e.s.d.'s is used for estimating e.s.d.'s involving l.s. planes.
Refinement. Refinement of *F*^2^ against ALL reflections. The weighted *R*-factor *wR* and goodness of fit *S* are based on *F*^2^, conventional *R*-factors *R* are based on *F*, with *F* set to zero for negative *F*^2^. The threshold expression of *F*^2^ > σ(*F*^2^) is used only for calculating *R*-factors(gt) *etc*. and is not relevant to the choice of reflections for refinement. *R*-factors based on *F*^2^ are statistically about twice as large as those based on *F*, and *R*- factors based on ALL data will be even larger.The methylene carbon atoms and the associated hydrogen atoms of the dioxine ring are disordered over two sites (O1—C5A—C6A—O2 and O1—C5B—C6B—O2) with occupancies of 0.36 (2):0.64 (2). The values were determined by refining site occupancies.

### Fractional atomic coordinates and isotropic or equivalent isotropic displacement parameters (Å^2^)

**Table:**

	*x*	*y*	*z*	*U*_iso_*/*U*_eq_	Occ. (<1)
S1	0.86744 (17)	0.05411 (9)	0.23931 (5)	0.0487 (3)	
O1	1.1659 (5)	0.5120 (3)	0.18957 (13)	0.0547 (6)	
O2	0.7713 (5)	0.4829 (2)	0.31477 (12)	0.0501 (5)	
O3	1.2360 (6)	0.0234 (3)	0.11029 (18)	0.0783 (8)	
O4	1.4165 (6)	0.2967 (3)	0.09719 (14)	0.0649 (7)	
O5	0.4735 (5)	−0.0297 (3)	0.36729 (15)	0.0632 (6)	
O6	0.4318 (4)	0.2289 (3)	0.39704 (12)	0.0491 (5)	
C1	1.0739 (6)	0.2137 (4)	0.19066 (17)	0.0430 (6)	
C2	1.0364 (6)	0.3648 (3)	0.21764 (16)	0.0400 (6)	
C3	0.8393 (6)	0.3508 (3)	0.27903 (15)	0.0377 (6)	
C4	0.7311 (6)	0.1889 (3)	0.29702 (16)	0.0399 (6)	
C5A	1.162 (5)	0.6525 (14)	0.2449 (14)	0.062 (5)	0.36 (2)
H5A1	1.2257	0.7562	0.2207	0.074*	0.36 (2)
H5A2	1.2966	0.6576	0.2893	0.074*	0.36 (2)
C6A	0.855 (5)	0.629 (2)	0.2697 (13)	0.059 (4)	0.36 (2)
H6A1	0.8417	0.7271	0.3011	0.070*	0.36 (2)
H6A2	0.7183	0.6125	0.2246	0.070*	0.36 (2)
C5B	1.018 (4)	0.6382 (11)	0.2131 (7)	0.065 (3)	0.64 (2)
H5B1	0.8276	0.6153	0.1843	0.078*	0.64 (2)
H5B2	1.1379	0.7469	0.2017	0.078*	0.64 (2)
C6B	0.971 (4)	0.6381 (10)	0.2987 (7)	0.063 (3)	0.64 (2)
H6B1	1.1603	0.6543	0.3272	0.076*	0.64 (2)
H6B2	0.8898	0.7292	0.3153	0.076*	0.64 (2)
C7	1.2491 (7)	0.1675 (4)	0.12851 (19)	0.0517 (7)	
C8	1.5944 (10)	0.2624 (6)	0.0335 (2)	0.0806 (12)	
H8A	1.7836	0.3470	0.0367	0.097*	
H8B	1.6335	0.1552	0.0378	0.097*	
C9	1.4402 (13)	0.2613 (7)	−0.0398 (3)	0.1016 (16)	
H9A	1.2575	0.1739	−0.0439	0.152*	
H9B	1.5623	0.2426	−0.0809	0.152*	
H9C	1.3978	0.3666	−0.0435	0.152*	
C10	0.5316 (6)	0.1166 (3)	0.35600 (17)	0.0444 (6)	
C11	0.2365 (7)	0.1649 (4)	0.45733 (19)	0.0571 (8)	
H11A	0.3405	0.1178	0.4951	0.069*	
H11B	0.0649	0.0783	0.4352	0.069*	
C12	0.1406 (9)	0.3054 (5)	0.4950 (2)	0.0747 (11)	
H12A	0.3081	0.3833	0.5227	0.112*	
H12B	−0.0094	0.2637	0.5303	0.112*	
H12C	0.0612	0.3602	0.4562	0.112*	

### Atomic displacement parameters (Å^2^)

**Table:**

	*U*^11^	*U*^22^	*U*^33^	*U*^12^	*U*^13^	*U*^23^
S1	0.0565 (5)	0.0320 (4)	0.0588 (5)	0.0137 (3)	0.0039 (3)	0.0041 (3)
O1	0.0743 (14)	0.0356 (11)	0.0540 (13)	0.0104 (10)	0.0276 (10)	0.0088 (9)
O2	0.0703 (13)	0.0307 (10)	0.0490 (12)	0.0103 (9)	0.0232 (10)	0.0043 (8)
O3	0.0888 (18)	0.0575 (15)	0.095 (2)	0.0335 (14)	0.0233 (15)	−0.0121 (14)
O4	0.0793 (16)	0.0678 (16)	0.0562 (14)	0.0309 (13)	0.0280 (12)	0.0075 (12)
O5	0.0707 (14)	0.0373 (12)	0.0790 (17)	0.0050 (10)	0.0115 (12)	0.0213 (11)
O6	0.0515 (11)	0.0418 (11)	0.0513 (12)	0.0041 (9)	0.0154 (9)	0.0127 (9)
C1	0.0454 (15)	0.0401 (15)	0.0445 (16)	0.0144 (12)	0.0023 (12)	0.0004 (12)
C2	0.0462 (14)	0.0328 (13)	0.0396 (14)	0.0079 (11)	0.0048 (11)	0.0039 (11)
C3	0.0421 (13)	0.0317 (13)	0.0379 (14)	0.0079 (10)	0.0030 (10)	0.0028 (11)
C4	0.0429 (14)	0.0313 (13)	0.0444 (15)	0.0080 (11)	0.0017 (11)	0.0049 (11)
C5A	0.089 (10)	0.021 (4)	0.066 (9)	0.000 (5)	0.024 (7)	−0.007 (5)
C6A	0.089 (11)	0.029 (5)	0.059 (10)	0.016 (6)	0.017 (7)	0.004 (6)
C5B	0.109 (8)	0.036 (3)	0.056 (5)	0.023 (4)	0.038 (5)	0.013 (3)
C6B	0.100 (7)	0.026 (3)	0.055 (5)	0.002 (4)	0.036 (4)	0.004 (3)
C7	0.0530 (17)	0.0508 (18)	0.0554 (18)	0.0238 (14)	0.0030 (14)	−0.0049 (15)
C8	0.082 (3)	0.106 (3)	0.068 (3)	0.047 (2)	0.028 (2)	0.005 (2)
C9	0.154 (5)	0.099 (4)	0.071 (3)	0.066 (3)	0.027 (3)	0.006 (3)
C10	0.0441 (14)	0.0361 (14)	0.0488 (16)	0.0014 (11)	0.0009 (12)	0.0122 (12)
C11	0.0505 (17)	0.064 (2)	0.0530 (19)	0.0031 (15)	0.0139 (14)	0.0208 (16)
C12	0.073 (2)	0.079 (3)	0.062 (2)	0.003 (2)	0.0216 (18)	0.000 (2)

### Geometric parameters (Å, °)

**Table:**

S1—C4	1.716 (3)	C5A—H5A2	0.9700
S1—C1	1.720 (3)	C6A—H6A1	0.9700
O1—C2	1.352 (3)	C6A—H6A2	0.9700
O1—C5B	1.453 (8)	C5B—C6B	1.508 (18)
O1—C5A	1.466 (13)	C5B—H5B1	0.9700
O2—C3	1.345 (3)	C5B—H5B2	0.9700
O2—C6B	1.435 (9)	C6B—H6B1	0.9700
O2—C6A	1.468 (16)	C6B—H6B2	0.9700
O3—C7	1.207 (4)	C8—C9	1.439 (6)
O4—C7	1.319 (4)	C8—H8A	0.9700
O4—C8	1.463 (4)	C8—H8B	0.9700
O5—C10	1.213 (3)	C9—H9A	0.9600
O6—C10	1.329 (4)	C9—H9B	0.9600
O6—C11	1.451 (3)	C9—H9C	0.9600
C1—C2	1.373 (4)	C11—C12	1.483 (5)
C1—C7	1.468 (4)	C11—H11A	0.9700
C2—C3	1.425 (4)	C11—H11B	0.9700
C3—C4	1.376 (4)	C12—H12A	0.9600
C4—C10	1.463 (4)	C12—H12B	0.9600
C5A—C6A	1.48 (3)	C12—H12C	0.9600
C5A—H5A1	0.9700		
			
C4—S1—C1	91.98 (13)	H5B1—C5B—H5B2	108.2
C2—O1—C5B	111.5 (4)	O2—C6B—C5B	110.0 (11)
C2—O1—C5A	111.2 (6)	O2—C6B—H6B1	109.7
C5B—O1—C5A	33.1 (6)	C5B—C6B—H6B1	109.7
C3—O2—C6B	112.4 (4)	O2—C6B—H6B2	109.7
C3—O2—C6A	111.3 (7)	C5B—C6B—H6B2	109.7
C6B—O2—C6A	28.4 (6)	H6B1—C6B—H6B2	108.2
C7—O4—C8	117.3 (3)	O3—C7—O4	125.4 (3)
C10—O6—C11	115.5 (2)	O3—C7—C1	121.2 (3)
C2—C1—C7	131.7 (3)	O4—C7—C1	113.4 (3)
C2—C1—S1	111.6 (2)	C9—C8—O4	110.4 (3)
C7—C1—S1	116.7 (2)	C9—C8—H8A	109.6
O1—C2—C1	125.0 (3)	O4—C8—H8A	109.6
O1—C2—C3	122.6 (2)	C9—C8—H8B	109.6
C1—C2—C3	112.4 (2)	O4—C8—H8B	109.6
O2—C3—C4	124.8 (2)	H8A—C8—H8B	108.1
O2—C3—C2	122.8 (2)	C8—C9—H9A	109.5
C4—C3—C2	112.4 (2)	C8—C9—H9B	109.5
C3—C4—C10	131.5 (3)	H9A—C9—H9B	109.5
C3—C4—S1	111.6 (2)	C8—C9—H9C	109.5
C10—C4—S1	116.8 (2)	H9A—C9—H9C	109.5
O1—C5A—C6A	108.4 (18)	H9B—C9—H9C	109.5
O1—C5A—H5A1	110.0	O5—C10—O6	124.2 (3)
C6A—C5A—H5A1	110.0	O5—C10—C4	122.8 (3)
O1—C5A—H5A2	110.0	O6—C10—C4	112.9 (2)
C6A—C5A—H5A2	110.0	O6—C11—C12	107.9 (3)
H5A1—C5A—H5A2	108.4	O6—C11—H11A	110.1
O2—C6A—C5A	110.1 (18)	C12—C11—H11A	110.1
O2—C6A—H6A1	109.6	O6—C11—H11B	110.1
C5A—C6A—H6A1	109.6	C12—C11—H11B	110.1
O2—C6A—H6A2	109.6	H11A—C11—H11B	108.4
C5A—C6A—H6A2	109.6	C11—C12—H12A	109.5
H6A1—C6A—H6A2	108.2	C11—C12—H12B	109.5
O1—C5B—C6B	109.7 (11)	H12A—C12—H12B	109.5
O1—C5B—H5B1	109.7	C11—C12—H12C	109.5
C6B—C5B—H5B1	109.7	H12A—C12—H12C	109.5
O1—C5B—H5B2	109.7	H12B—C12—H12C	109.5
C6B—C5B—H5B2	109.7		
			
C4—S1—C1—C2	−0.5 (2)	C2—O1—C5A—C6A	−50 (3)
C4—S1—C1—C7	−179.3 (2)	C5B—O1—C5A—C6A	46.7 (17)
C5B—O1—C2—C1	162.8 (8)	C3—O2—C6A—C5A	−48 (2)
C5A—O1—C2—C1	−161.5 (13)	C6B—O2—C6A—C5A	50 (2)
C5B—O1—C2—C3	−16.3 (9)	O1—C5A—C6A—O2	67 (3)
C5A—O1—C2—C3	19.4 (13)	C2—O1—C5B—C6B	47.5 (16)
C7—C1—C2—O1	−0.2 (5)	C5A—O1—C5B—C6B	−48.7 (13)
S1—C1—C2—O1	−178.7 (2)	C3—O2—C6B—C5B	46.3 (17)
C7—C1—C2—C3	179.0 (3)	C6A—O2—C6B—C5B	−47.3 (18)
S1—C1—C2—C3	0.5 (3)	O1—C5B—C6B—O2	−65 (2)
C6B—O2—C3—C4	164.7 (8)	C8—O4—C7—O3	1.9 (5)
C6A—O2—C3—C4	−164.7 (11)	C8—O4—C7—C1	−178.7 (3)
C6B—O2—C3—C2	−14.9 (9)	C2—C1—C7—O3	−174.9 (3)
C6A—O2—C3—C2	15.7 (11)	S1—C1—C7—O3	3.5 (4)
O1—C2—C3—O2	−1.3 (4)	C2—C1—C7—O4	5.7 (5)
C1—C2—C3—O2	179.5 (2)	S1—C1—C7—O4	−175.8 (2)
O1—C2—C3—C4	179.0 (3)	C7—O4—C8—C9	95.4 (4)
C1—C2—C3—C4	−0.2 (4)	C11—O6—C10—O5	−1.4 (4)
O2—C3—C4—C10	−1.2 (5)	C11—O6—C10—C4	−179.1 (2)
C2—C3—C4—C10	178.4 (3)	C3—C4—C10—O5	−175.0 (3)
O2—C3—C4—S1	−179.9 (2)	S1—C4—C10—O5	3.6 (4)
C2—C3—C4—S1	−0.2 (3)	C3—C4—C10—O6	2.7 (5)
C1—S1—C4—C3	0.4 (2)	S1—C4—C10—O6	−178.76 (18)
C1—S1—C4—C10	−178.4 (2)	C10—O6—C11—C12	−178.2 (3)

### Hydrogen-bond geometry (Å, °)

**Table:**

*D*—H···*A*	*D*—H	H···*A*	*D*···*A*	*D*—H···*A*
C9—H9B···O3^i^	0.96	2.66	3.333 (5)	127
C9—H9A···O3^ii^	0.96	2.62	3.523 (7)	157
C6B—H6B1···O5^iii^	0.97	2.68	3.233 (12)	117

Symmetry codes: (i) −*x*+3, −*y*, −*z*; (ii) −*x*+2, −*y*, −*z*; (iii) *x*+1, *y*+1, *z*.
